# AI-driven estimation of O6 methylguanine-DNA-methyltransferase (MGMT) promoter methylation in glioblastoma patients: a systematic review with bias analysis

**DOI:** 10.1007/s00432-023-05566-5

**Published:** 2024-01-31

**Authors:** Mullapudi Venkata Sai Samartha, Navneet Kumar Dubey, Biswajit Jena, Gorantla Maheswar, Wen-Cheng Lo, Sanjay Saxena

**Affiliations:** 1 Department of Computer Science & Engineering, International Institute of Information Technology, Bhubaneswar, 751003 India; 2 Victory Biotechnology Co., Ltd., Taipei, 114757 Taiwan; 3 Executive Programme in Healthcare Management, Indian Institute of Management, Lucknow, 226013 India; 4 Institute of Technical Education and Research, SOA Deemed to be University, Bhubaneswar, 751030 India; 5 Division of Neurosurgery, Department of Surgery, School of Medicine, College of Medicine, Taipei Medical University, Taipei, 11031 Taiwan; 6 Department of Neurosurgery, Taipei Medical University Hospital, Taipei, 11031 Taiwan; 7 Taipei Neuroscience Institute, Taipei Medical University, Taipei, 11031 Taiwan

**Keywords:** O(6)-methylguanine-DNA-methyltransferase (MGMT), Methylation status, Radiogenomics, Artificial intelligence (AI), Machine learning, Deep learning

## Abstract

**Background:**

Accurate and non-invasive estimation of MGMT promoter methylation status in glioblastoma (GBM) patients is of paramount clinical importance, as it is a predictive biomarker associated with improved overall survival (OS). In response to the clinical need, recent studies have focused on the development of non-invasive artificial intelligence (AI)-based methods for MGMT estimation. In this systematic review, we not only delve into the technical aspects of these AI-driven MGMT estimation methods but also emphasize their profound clinical implications. Specifically, we explore the potential impact of accurate non-invasive MGMT estimation on GBM patient care and treatment decisions.

**Methods:**

Employing a PRISMA search strategy, we identified 33 relevant studies from reputable databases, including PubMed, ScienceDirect, Google Scholar, and IEEE Explore. These studies were comprehensively assessed using 21 diverse attributes, encompassing factors such as types of imaging modalities, machine learning (ML) methods, and cohort sizes, with clear rationales for attribute scoring. Subsequently, we ranked these studies and established a cutoff value to categorize them into low-bias and high-bias groups.

**Results:**

By analyzing the 'cumulative plot of mean score' and the 'frequency plot curve' of the studies, we determined a cutoff value of 6.00. A higher mean score indicated a lower risk of bias, with studies scoring above the cutoff mark categorized as low-bias (73%), while 27% fell into the high-bias category.

**Conclusion:**

Our findings underscore the immense potential of AI-based machine learning (ML) and deep learning (DL) methods in non-invasively determining MGMT promoter methylation status. Importantly, the clinical significance of these AI-driven advancements lies in their capacity to transform GBM patient care by providing accurate and timely information for treatment decisions. However, the translation of these technical advancements into clinical practice presents challenges, including the need for large multi-institutional cohorts and the integration of diverse data types. Addressing these challenges will be critical in realizing the full potential of AI in improving the reliability and accessibility of MGMT estimation while lowering the risk of bias in clinical decision-making.

## Introduction

Glioblastoma multiforme (GBM), is a malignant and aggressive brain tumor that spreads aggressively (Jena et al. 2022) and typically originates in the adult cerebrum, the brain's largest region (Peri 2022). The median survival time in GBM patients is approximately nine months; however, those with standard-of-care surgery and adjuvant chemoradiation may extend to 15–16 months (Brain tumor segmentation and overall survival period prediction in glioblastoma multiforme using radiomic features-Das 2022; Tamimi and Juweid 2017). The economic burden has been prominently evidenced among the affected individuals receiving systemic medication (Raizer et al. 2015; Kumthekar et al. 2014). Neurological examinations and neuroimaging techniques are the primary diagnostic tools for GBM identification but could be employed only after the disease has significantly progressed. It is often treated with surgery to remove the tumour mass, followed by radiotherapy and chemotherapy. Regardless of the surgery's extent, GBM resection is frequently insufficient, resulting in relapse or even recurrence (Silantyev et al. 2019). Various imaging modalities are employed, such as magnetic resonance imaging (MRI), computer tomography (CT), digital subtraction angiography (DSA), and to a certain extent, even X-Rays for non-invasive determination of GBM. Although there are multiple neuro-imaging paradigms, MRI is preferred to other modalities for various reasons, including its ability to image discrete anatomical regions in arbitrary planes with excellent tissue contrast and the lack of evident negative health impacts on patients (An empirical study of different machine learning techniques for brain tumor classification and subsequent segmentation using hybrid texture feature|SpringerLink 2022).

O6-methylguanine-DNA methyltransferase (MGMT), a DNA-repairing enzyme, is located on the 10q26.3 chromosome (Methylguanine-DNA methyltransferase (MGMT)|Radiology Reference Article|Radiopaedia.org 2022). High MGMT activity reduces the effectiveness of alkylating drugs and is a poor prognostic indicator. However, when the MGMT promoter is methylated, alkylating agents are more potent (Methylguanine-DNA methyltransferase (MGMT)|Radiology Reference Article|Radiopaedia.org 2022**).** It has been examined as a potential biomarker of susceptibility to alkylating chemotherapy, particularly temozolomide (TMZ), because of its relatively high frequency in GBM, which may vary depending on the method employed for its assessment (Stupp et al. May 2009). In a study by Hegi et al. (MGMT Gene Silencing and Benefit from Temozolomide in Glioblastoma|NEJM”. 2022), the authors stated that “Among patients whose tumor contained a methylated *MGMT* promoter, a survival benefit was observed in patients treated with temozolomide and radiotherapy; “their median survival was 21.7 months as compared with 15.3 months among those who were assigned to only radiotherapy. In the absence of methylation of the *MGMT* promoter, there was a smaller and statistically insignificant difference in survival between the treatment groups”.

In current clinical practice in the era of precision medicine, invasive methods such as biopsy and surgery are the only reliable ways for MGMT methylation status determination. However, such invasive methods carry threats and difficulties, for example, neurologic injury, complications, cost, etc. Hence, researchers have been working on AI-based methods using medical imaging modalities for MGMT status determination in the last couple of years. The idea of AI is to strive to use computers to imitate human intelligence. This field has much potential in radiological-based medical applications (Jena et al. 2021). Deep Learning (DL) is a paradigm of AI wherein the programmer tries to create a mathematical model mimicking the human mind called Neural Networks (NN). These DL models, coupled with the traditional Machine Learning (ML) models, possess the potential to detect the methylation status of the MGMT gene, among other biomarkers, without needing a biopsy directly from the neuro-image sequences. In a study by Xi et al. (2018) Support Vector Machines (SVMs) have been used to analyze radiomics features for utilizing the full potential of medical imaging as biomarkers of MGMT promoter methylation. The results revealed an accuracy of 86.59% using the T1, T2, and enhanced T1CE image features of the MRI scans of the 98 GBM patients. This study stated that there is a space for the ML models to churn the neuro-imaging data into a viable option for biopsy. DL models, too, are showing promising results. Chen et al. (2020) devised a DL pipeline to automate the prediction of MGMT status. They have considered 106 GBM patients with contrast-enhanced T1W images and fluid-attenuated inversion recovery (FLAIR) images. Using a pipeline model consisting of both tumor segmentation and classification, they have concluded that their pipeline best works on FLAIR images with an 82.7 ± 5.6% accuracy. Their suggested pipeline reduced inter-observer variation in glioma segmentation, sped the tumour annotation process, and accurately predicted the MGMT methylation status. It would make finding molecular biomarkers from common medical imaging even easier. There is constant innovation in models designed to make the results indistinguishable from the biopsy ones.

In this study, we conducted a systematic review and analysis of 33 research articles on estimating MGMT promoter methylation using various AI methodologies and its ML and DL components. Thereafter, we have presented the recent developments in the contribution of AI as per the neurological perspective of MGMT methylation. Eventually, the bias analysis was performed on the selected studies, and principal findings and challenges were discussed.

## Search strategy and statistics

### Preferred reporting items for systematic reviews and meta-analyses (PRISMA) model

**Fig. 1:**
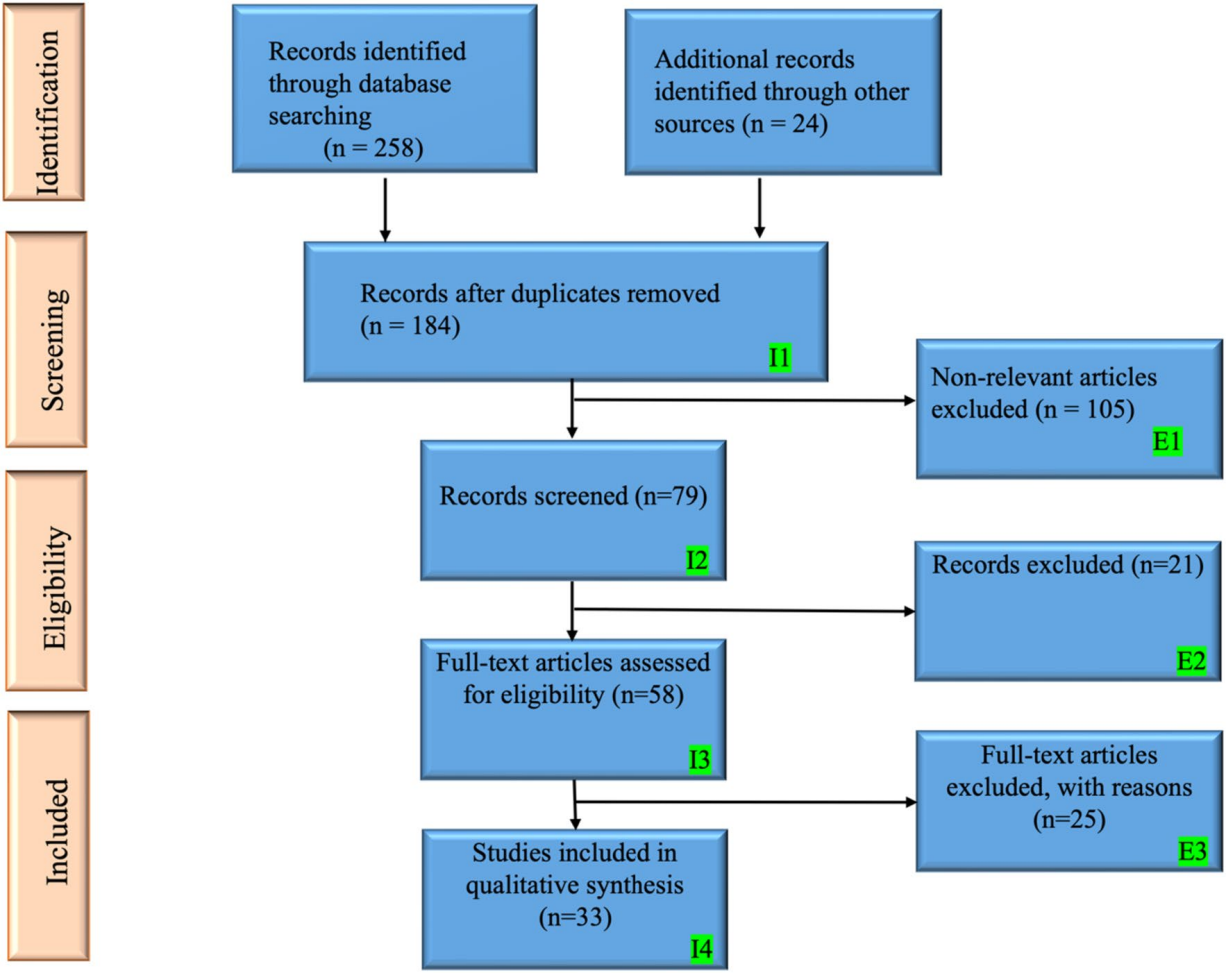
The PRISMA model. I: Inclusion criteria, E: Exclusion criteria

We conducted an extensive literature search on *PubMed, ScienceDirect, Google Scholar,* and *IEEE Explore* using the PRISMA strategy (Fig. 1). The keywords included were: (Glioblastoma AND MGMT AND Machine Learning AND Artificial Intelligence), (Gliomas AND MGMT AND Machine Learning AND Deep Learning), and (MGMT AND (Machine Learning OR Deep Learning OR Artificial Intelligence OR Radiomics OR Radiogenomics) AND (gliomas OR glioblastomas)).

Using Clarivate Analytics' EndNote software's "Find Duplicates" option, a maximum of 258 papers were found and replicas were eliminated, leaving 184 entries. Studies unrelated to AI, irrelevant papers, and articles with inadequate data were the three exclusion criteria. The final 33 references for this study were chosen after applying the exclusion criteria to 105, 21, and 25 studies (designated as E1, E2, and E3 in Fig. 1), which were then located and discarded.

### Statistical distribution and analysis based on various parameters

#### Dataset size (DS)

**Fig. 2:**
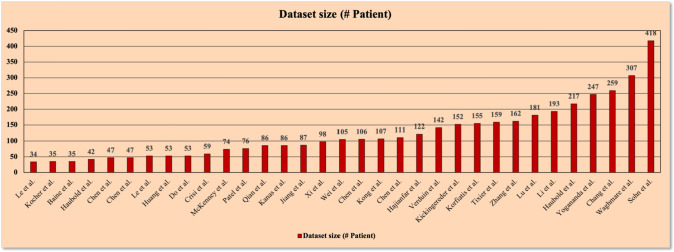
The distribution of cohort size in several trials for AI-based MGMT detection

We observed that the data set size ranged from 34 to 418 patients across the 33 publications. DS is the total number of patients used to capture images in MRI, CT, PET, or a combination of these three modalities. The distribution of the dataset in various studies is displayed in Fig. 2.

#### Studies with AI application

**Fig. 3:**
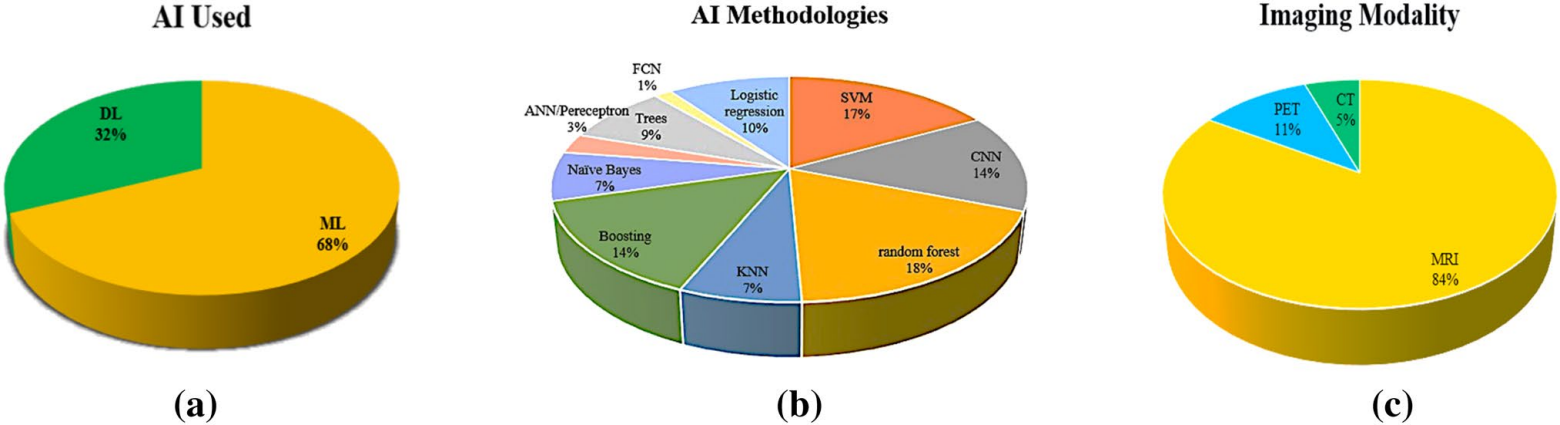
**a**. Distribution of AI applications (ML and DL) across various studies. *ML* machine learning, *DL* deep learning. **b** Application of different AI methodologies for detecting MGMT status. *ANN* artificial neural network, *CNN* Convolutional neural network, *kNN* k-nearest neighbors, *SVM* Support vector machines, *FCN* Fully Convolutional Network. **c** Various imaging modalities are used in datasets. *MRI* magnetic resonance imaging, *CT* computed tomography, *PET* Positron emission tomography

Figure 3a shows the prevalence of machine learning (ML) and deep learning (DL) methodologies. Most of the studies employed the ML methods rather than DL. This may be attributed to DL models requiring a good-quality dataset which is easily accessible.

#### AI methodologies

Though various modalities have been used to accurately detect MGMT status, the most prominent ones include Random Forests and Vector Machines. It has also been revealed that compared to individuals, the combination of modalities may be effective (Stupp et al. 2009). Moreover, DL models have higher accuracies than simple ML models. To detect MGMT status, various AI methodologies have been represented in Fig. 3b.

#### MRI—an efficient imaging modality to analyze GBM

MRI is a frequently employed technique that creates three-dimensional, intricate anatomical images that help diagnose disease and monitor therapy. It detects changes in the rotational axis of protons in the water present in the living tissues (Magnetic Resonance Imaging (MRI) 2022). MRI plays a significant role in AI-based analysis. It can scan discrete anatomical locations in vivo with excellent tissue contrast with images that can be taken in any plane. Though CT and PET modalities are also very informative but are used to a lesser extent, the distribution of which has been represented in Fig. 3c.

## GBM and MGMT

### Glioma’s pathology: the WHO grading system

Glioma is a type of primary tumor that originates in the brain and spinal cord and initiates in the gluey supportive cells (glial cells) surrounding nerve cells (Stupp et al. 2009). Due to its complex nature, gliomas are often referred to as intra-axial brain tumors. The prognosis and course of therapy are influenced by the type of glioma, and the treatment options include surgical, radiation therapy, chemotherapy, and targeted therapy.

**Fig. 4:**
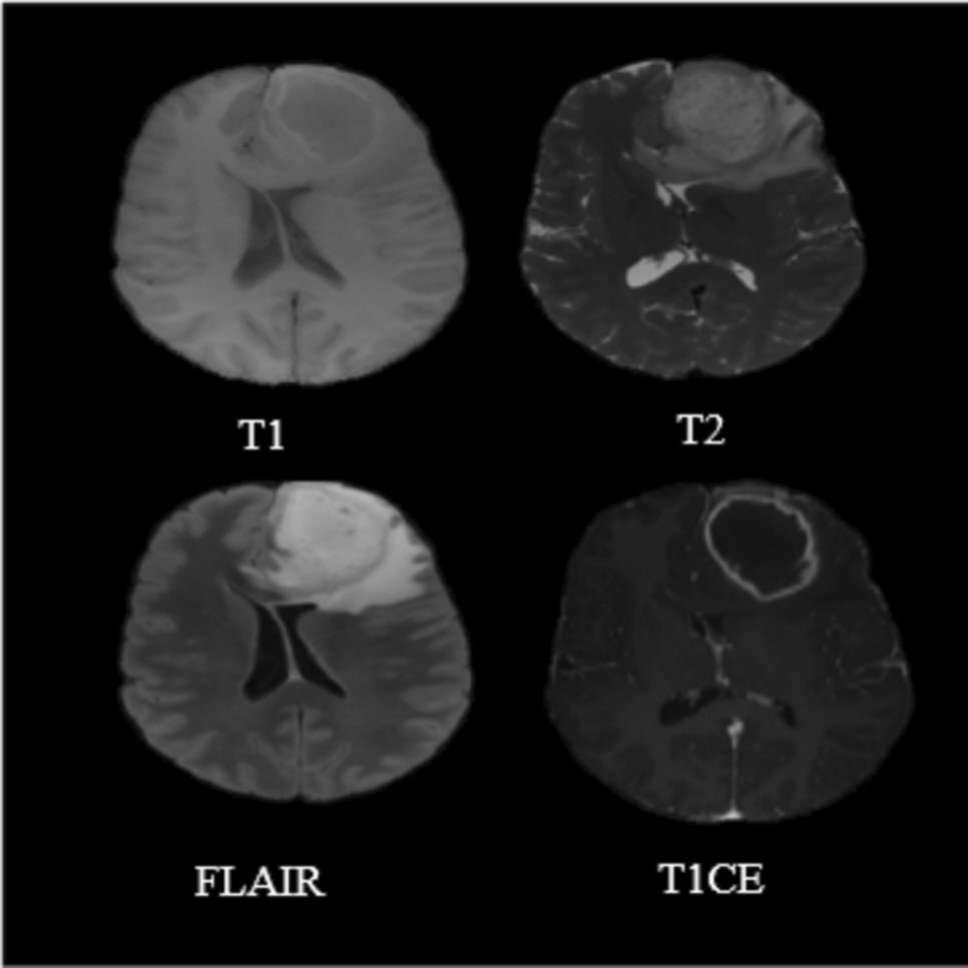
Structural MR (T1, T2, FLAIR and T1CE(or T1GD) scans of GBM Patients

Astrocytomas, ependymomas, and oligodendrogliomas are the three primary forms of gliomas, which are categorized based on phenotypic cell features (Magnetic Resonance Imaging (MRI) 2022). These cell gliomas are further divided into low-grade, atypical, and high-grade tumors based on cell morphology, mitotic activities, and molecular marker. The World Health Organization (WHO) grading system recommends molecular markers with proven prognostic and therapeutic implications. For example, GBM is a type of glioma that has progressed to the fourth-grade (Lopes Oct. 2017; Mesfin and Al-Dhahir 2022). Figure 4 shows an MR scan of the GBM-affected brain, and Table 1 shows the histologic type and grade of glioma.

**Table 1:** Glioma histologic Grading (Rasmussen et al. 2017; New Strategies Take on the Worst Cancer-Glioblastoma-Scientific American 2022)

WHO grade	Histologic type
GRADE I	Subependymal giant-cell astrocytoma, Pilocytic astrocytoma
GRADE II	Oligoastrocytoma, Diffuse astrocytoma, Gemistocytic astrocytoma, Pleomorftxanthoastrocytoma, Oligodendroglioma
GRADE III	Gliomatosis cerebri, Anaplastic oligoastrocytoma, Anaplastic astrocytoma, Anaplastic oligodendroglioma
GRADE IV	Glioblastoma, Glioblastoma with sarcomatosis

### MGMT in GBM

**Fig. 5:**
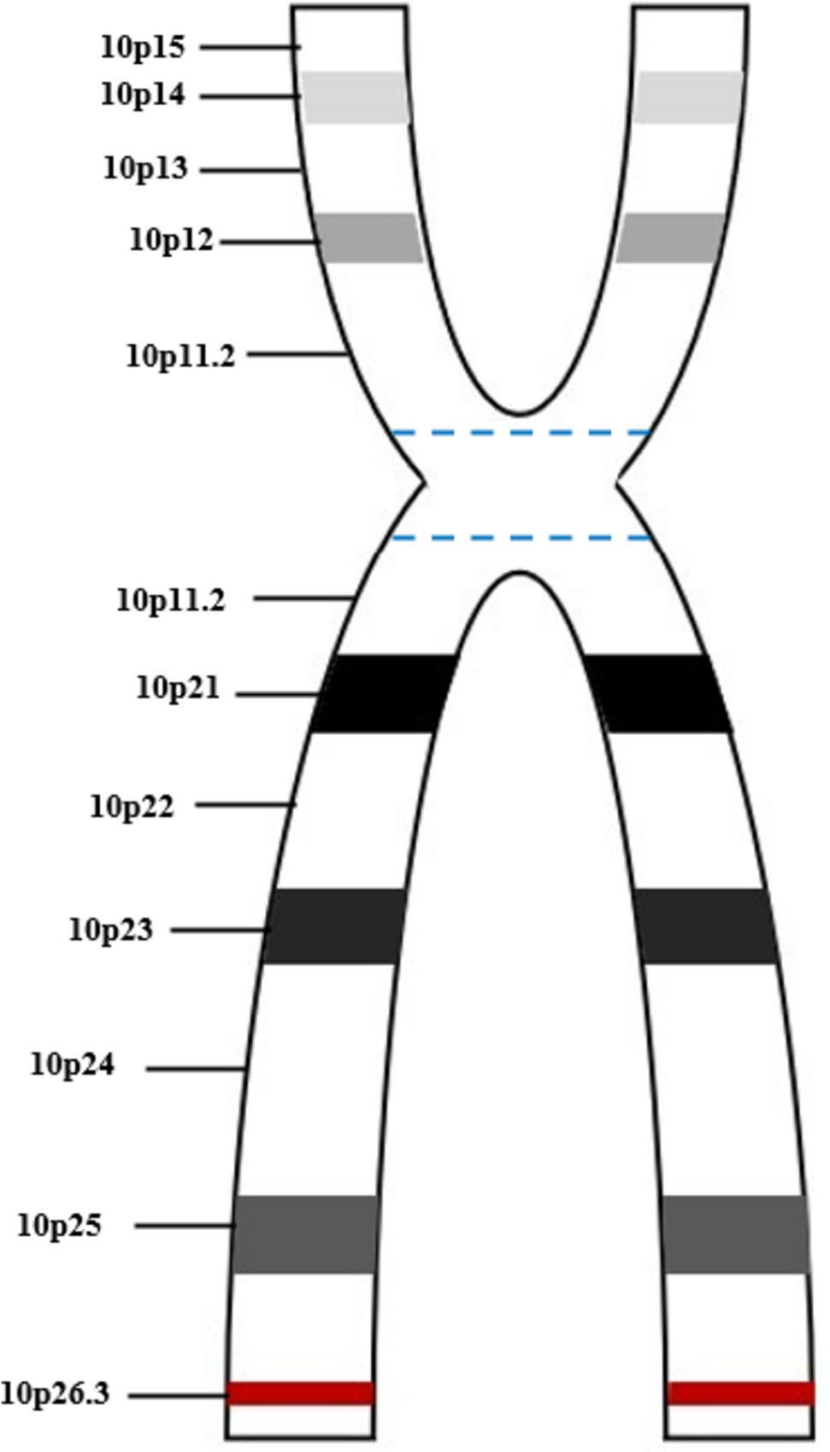
Representative diagram showing the MGMT gene located on chromosome 10q26.3 (Yu et al. 2020)

MGMT is a DNA "suicide" repair enzyme. Transfer of methyl group from guanine's O6 site to its cysteine residues restores damaged guanine nucleotides without causing gene mutation, cell death, or tumorigenesis from alkylating agents (Gerstner et al. 2009). MGMT gene is located on chromosome 10q26.3 (Fig. 5), with a total length of 300,437 bp (Yu et al. 2020). Methylation of the MGMT gene promoter significantly predicts prognosis for newly diagnosed GBM. MGMT has recently been linked to the therapeutic success of alkylating agent chemotherapy, specifically temozolomide (TMZ) treatment (Sharma et al. 2009). It is commonly believed that MGMT promoter methylation in patient tumors results in reduced MGMT protein production, and elimination of the DNA repair activity required for TMZ resistance as MGMT transcription may be repressed by promoter methylation in tumor cells (Brandes et al. 2017), according to Liu et al. (2006) and Pistollato et al. (2010), GBM stem cells, which the stem cell marker CD133 can recognize, express a high amount of MGMT and have significant tumor resistance to TMZ. According to CD133(-) glioblastoma-derived cancer stem cells show differential growth characteristics and molecular profiles-PubMed (2022), Beier et al. (2008), several stem cell types exhibit different MGMT protein expressions despite having equivalent MGMT promoter methylation status. It was further discovered that TMZ selectively destroys glioblastoma cancer stem cells in MGMT-negative cell lines, indicating this protein's potential in cancer treatment. The MGMT levels have been successfully manipulated to not only improve alkylating agent therapy but also to safeguard hematopoietic cells from the myelosuppressive effects of high-dose chemotherapy (Sharma et al. 2009).

## Role of AI in current clinical practice

### Recent development in predicting MGMT status.

Till now, numerous studies have demonstrated that MGMT promoter methylation is a significant predictive biomarker for TMZ resistance and poor progression-free survival in GBM patients (Yin et al. 2014; Gerstner et al. 2009; Butler et al. 2020; OncologyPRO 2019; Saxena et al. 2023a; Saxena et al. 2023b; Sareen et al. 2022). Methylation-specific polymerase chain reactions using surgical specimens are considered the gold standard for evaluating the MGMT methylation status; however, they require a large volume of tissue samples and strict sample cryopreservation procedures (Stupp et al. 2009). Other techniques, such as activity assays, immunohistochemistry, and methylation chip analysis, have technical limitations (Drabycz et al. 2010). These invasive procedures are also less helpful in hospitals due to the potential of insufficient biopsy samples, expensive detection costs, and the great complexity of the intralesional heterogeneity (18F-FDG-PET-based Radiomics signature predicts MGMT promoter methylation status in primary diffuse glioma|Cancer Imaging|Full Text 2022).

As discussed in recent decades, experts have switched to finding correlations between clinical symptoms and genetic traits utilizing non-invasive methods like radiomics (A Deep Learning-Based Radiomics Model for Prediction of Survival in Glioblastoma Multiforme-PubMed 2022) to quantitatively extract and evaluate various noninvasive image data, including intensity distributions, spatial relationships, and patterns of textural heterogeneity (McGarry et al. 2016). And it is noticed the developments of radiomics models in radiology for predicting survival rates, distant metastasis, and molecular characterization (Kickingereder et al. 2016). In addition, numerous computer models were created to preoperatively predict the MGMT methylation status based on magnetic resonance imaging since it is thought that the MGMT methylation status is a significant predictive indicator for guiding GBM treatment decisions (MRI) (Xi et al. 2018; Li et al. 2018; Wei et al. 2019). Le et al. (2020), recently proposed a radiomics-based eXtreme Gradient Boosting (XGBoost) model that demonstrated reasonably good performance for predicting the MGMT promoter methylation status, with an accuracy of 88.7% and an area under the receiver operating characteristics curve (AUC) of 0.896. Do et al. (Improving MGMT methylation status prediction of glioblastoma through optimizing radiomics features using genetic algorithm-based machine learning approach|Scientific Reports 2022), suggested a hybrid ML-based radiomics feature selection model to find the best radiomics feature sets and predict the MGMT promoter methylation status in response to the earlier work of Le et al. (2020). Most of the radiomics feature sets for categorizing MGMT methylation statuses provided by other studies were based only on one feature selection technique. This study is the first to use the genetic algorithm-based hybrid feature selection approach for classifying MGMT methylation statuses in GBM.

To identify a radiomics feature subset that could accurately predict the MGMT methylation statuses, their study set out to explore the viability of adopting a two-stage feature selection approach composed of feature selection carried out using the XGBoost algorithm followed by a GA wrapper model (GA wrapper is a feature selection mechanism where each feature is considered as a gene and a selected set of features as a chromosome). They noticed that the implementation of the GA resulted in a radiomics feature set, which displayed greater accuracy levels for MGMT methylation status prediction than most of those reported in prior research. Additionally, their findings demonstrated that a smaller degree of prediction accuracy might be caused by either the inclusion of too few features (F-score feature set) or too many features. As a result, the GA provides a viable method for producing highly effective predictors without knowing the ideal number of features to be included in advance. This model with the highest performance (GA-RF) was tested on an independent dataset, which demonstrated that the model might be generalized to similar diseases. This cutting-edge model's ability to predict MGMT methylation status might benefit clinical decision-making by allowing for treatment strategies for patients with GBM even before surgery.

### Challenges and opportunities

Despite the enormous promise of AI in tumor diagnosis, prognosis, and prediction, translations into clinical settings are delayed because of several related difficulties (Ak et al. 2022). These substantial obstacles must be addressed to incorporate AI methods into healthcare settings. A key problem in predicting MGMT status using AI is the interpretation of the algorithms, which are exceedingly complicated. Interpreting their inner workings is not straightforward; it is called a 'black box' nature (Cuocolo et al. 2020). This makes it harder for such technologies to be used in healthcare. An algorithm that is simple to understand enables evaluation of its results and offers suggestions for improvement. Although important, these algorithms rely heavily on available data interpretation standards, which can also introduce bias (Elmore et al. 2016). The findings of these algorithms have consistently outperformed human readings regarding reproducibility and consistency; however, this leads to additional patient exams and overdiagnosis (Cuocolo et al. 2020).

The most challenging task in the next step is storing, managing, extracting, analyzing, integrating, visualizing, and communicating the information produced from the vast amount of accessible data (Pinta et al. 2021). Integrating such diverse and multivariate data in an economical, standardized, and safe way is crucial. Critical ongoing problems also include the nature and variability of the data. Despite the ease with which large amounts of imaging data are accessible, institutional heterogeneity (either intra- or inter) exists due to variations in scan protocols, technology, and post-processing procedures, which restricts the generalizability of findings (Pinta et al. 2021). In addition, there are variations in contrast enhancement procedures, arguments, and image acquisition settings. According to research, radiomic feature estimations still varied even when the identical scanning methodology was used for image acquisition. As a result, findings are less easily repeatable, hindering useful AI models' creation (Ger et al. 2018).

Another key problem connected with AI research is the restricted number of laboratories performing such research due to the costs and difficulties involved (Trivizakis et al. 2020). Furthermore, one critical issue of implementing AI is the need for appropriate nested cross-validation to minimize overfitting, which is typical in AI (Saxena et al. 2022). Finally, data on MGMT promoter status were only available for a selected patient subgroup of an overall trial population which can induce selection bias in the analysis (Yin et al. 2014).

### AI in MGMT status prediction: a neuroimaging perspective

This section presents neuro-imaging perspectives on the recent advancements in the methods of MGMT methylation estimation under the artificial intelligence paradigm. Radiological scans have proved an effective non-invasive technique for early-stage MGMT prediction in patients suffering from GBM with screening and diagnosis, support for treatment regimens, prognosis evaluation, and follow-up for advanced-stage of glioblastoma (Jena et al. 2022; Saxena et al. 2022). Recent years have seen the evolution of radiological features from semantic to radiomic hand-crafted and deep features. *Semantic features* are the qualitative characteristics that a skilled radiologist will typically derive from the clinical imaging directly to describe the lesion (An empirical study of different machine learning techniques for brain tumor classification and subsequent segmentation using hybrid texture feature|SpringerLink 2022; Rizzo et al. 2018). To examine potential connections with biology and clinical outcomes, *radiomic features*, on the other hand, comprise extracting and evaluating quantitative information from medical images using mathematical algorithms, machine learning, and deep learning techniques. The anatomical and functional knowledge of MGMT genomics can be separately reflected by radiomic characteristics retrieved from structural and functional imaging (Srivastava et al. 2019).

**Fig. 6:**
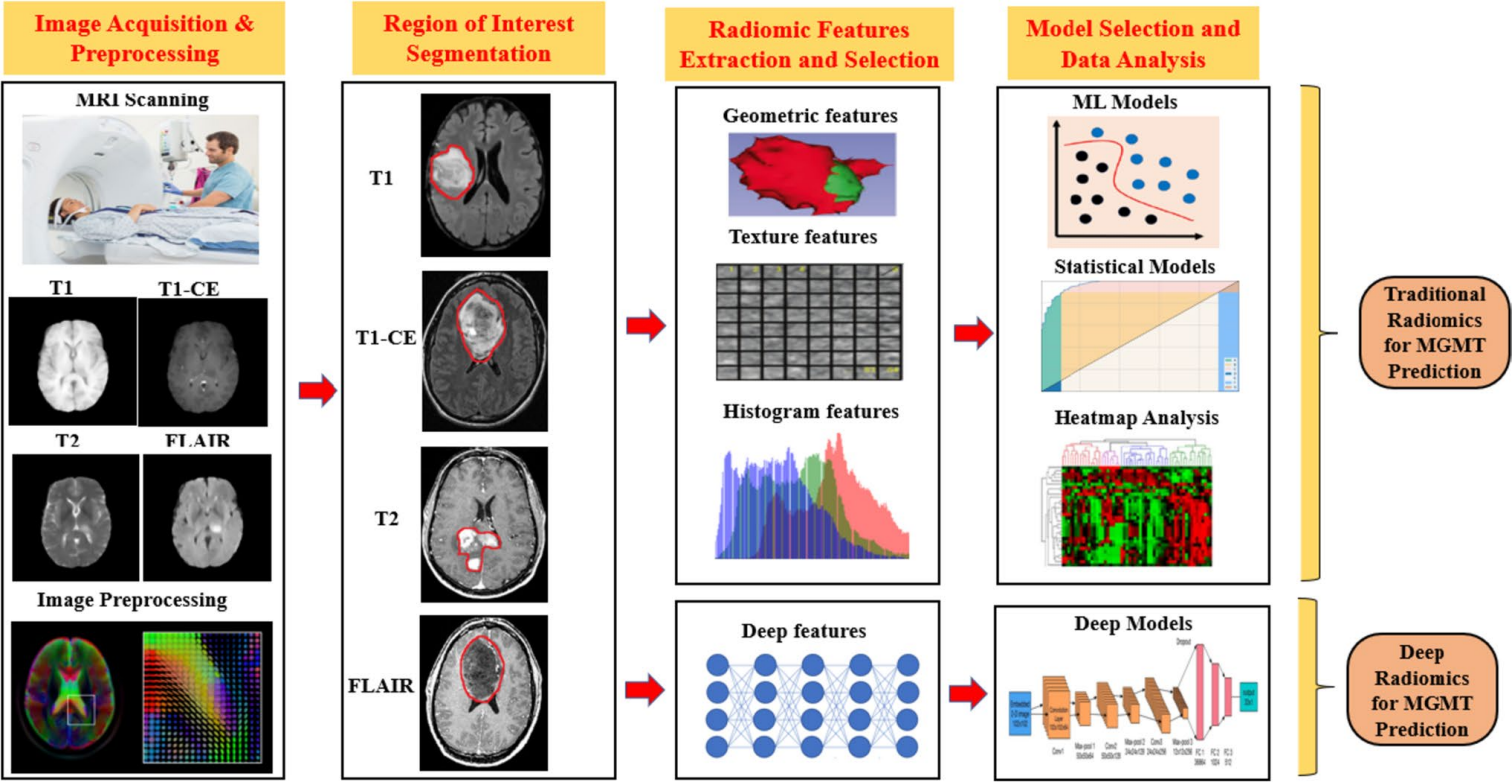
Complete Pipeline showing the MGMT promoter methylation status prediction with neuroimaging prospect under artificial intelligence paradigms. This pipeline includes image acquisition with different preprocessing steps, image segmentation, numerous features extraction and the development of various ML and DL models

Radiomics can be coupled with artificial intelligence (AI) due to its superior capacity to handle vast amounts of data when compared to conventional statistical methods. Together, these fields' main goal is to unearth and meaningfully evaluate as much buried quantitative data as possible for use in decision support. Due to their impressive accomplishment in a variety of radiological tasks, both radiomics and AI have recently attracted the attention (Radiomics with artificial intelligence: a practical guide for beginners-PMC 2022). The traditional radiomics workflow uses an AI framework. It includes a number of processes, such as image acquisition, pre-processing, region of interest segmentation, feature extraction, feature selection, model selection, evaluation, and validation with clinical implementation. Deep radiomics research is a part of deep learning (DL) technology, a branch of computer learning (ML). Image pre-processing is essential when dealing with clinical images with essential genomics information, such as MGMT, both for traditional and deep radiomics. Following pre-processing, the region of interest (ROI) has been identified, and radiomics features that contain genomics information have been retrieved from it. The radiomics feature may be hand-crafted or deep. The final step of the radiomics process is the model selection and data analysis from the radiomics feature for better clinical decision and treatment planning, as shown in Fig. 6.

## Risk-of-bias (RoB) analysis

**Fig. 7:**
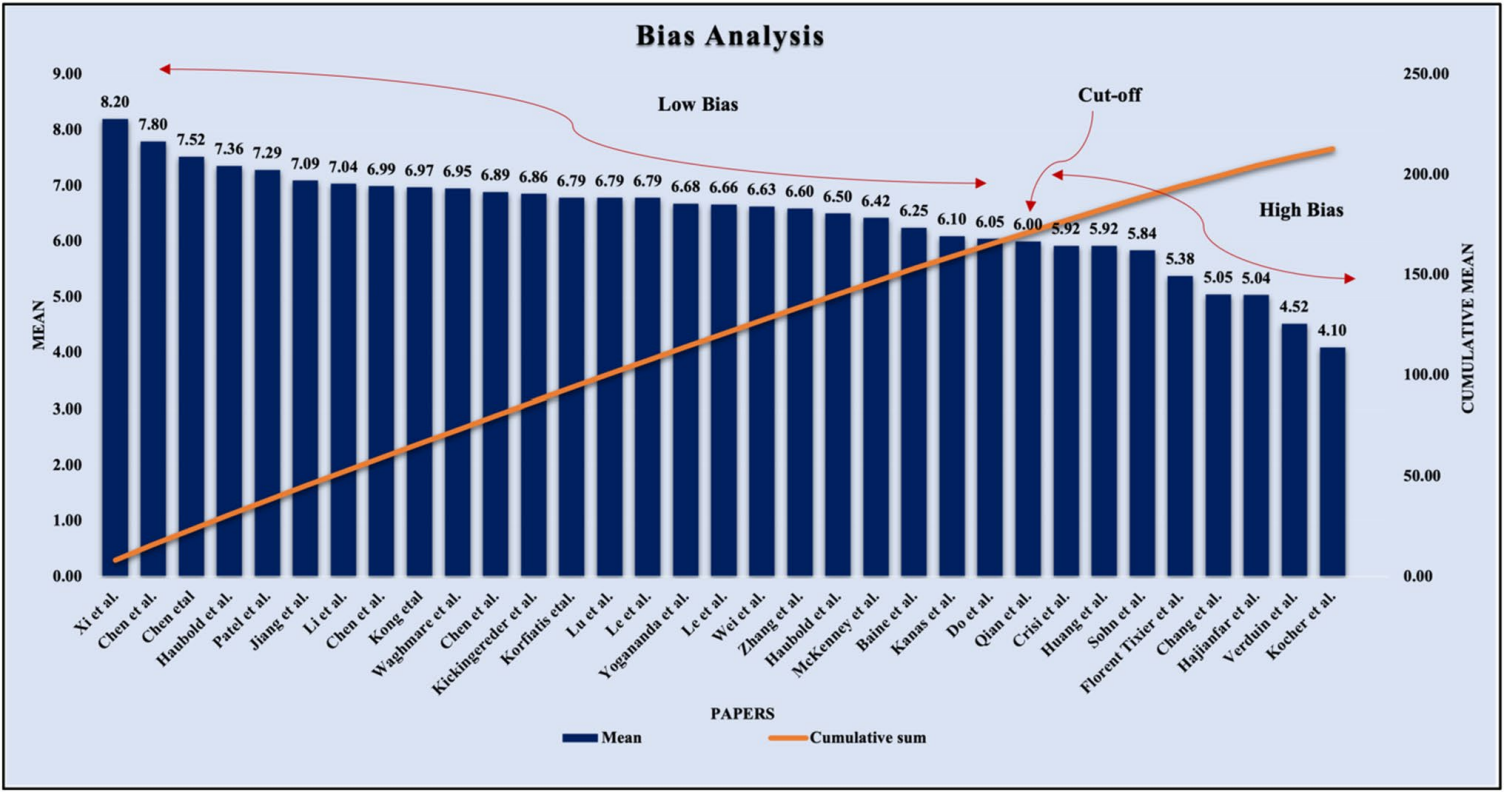
The ranking score technique shows the frequency distribution of radiogenomics studies for MGMT methylation in descending order, succeeded by the cumulative plot, showing the raw cut-off mark for bias analysis

As mentioned before, by PRISMA strategy, we considered 33 studies for MGMT methylation using AI and its components, such as ML and DL. Moreover, we performed RoB analysis to check the bias in these studies to show that AI is viable for MGMT methylation status determination and analysis in GBMs. Each study has been analyzed on 21 AI-based attributes such as image modalities, the objective of the study, the dataset size (in the number of patients), patients demography, feature extraction and selection, data preprocessing and augmentation, performance evaluation parameters like accuracy, sensitivity, specificity, precision, AUC of the ROC, the F-score, performance analysis metrics like the confusion matrix and ROC, statistical analysis, regularization, hardware and software resources used. These attributes using AI features are initially qualitative and then quantified by assigning a number between 0 and 1 based on the consensus of the AI scientist’s experience. The value of AI-based attributes has been set based on the attribute's strength, which ranges from 0 to 1. Then each study’s aggregate score is the sum of all attribute values for that selected study. The mean of each study was then calculated by dividing by the number of AI attributes considered (i.e., 21 in our case). Using this principle, all 33 studies are ranked based on their mean scores, ranging from 0.820 to 0.410. We multiplied all the mean values with 10 to normalize the scores between 1 and 10, then plotted them in decreasing order (Fig. 7). The raw cut-off of 6.00 was determined based on the intersection of the “cumulative plot of the mean score” and “the frequency plot curve of the studies”. This raw cut-off mark estimates the whole number of studies into low-bias and high-bias categories. The higher the mean value, the lower the risk of bias; hence, studies above the cut-off mark belong to the low-bias category, while 27% belong to the high-bias category. The highly biased studies have not considered all AI attributes while evaluating the radiogenomics system or may have low proportioned values for the attributes considered.

## Discussion

### Principal findings

As per the best of our rigorous search and findings, this is the first study of its kind to demonstrate AI approaches in predicting the MGMT status in GBM and the most recent developments in its prediction. The PRISMA methodology, a well-established benchmark in the healthcare business, was used to identify 33 studies. The investigation revealed several statistical distributions based on many criteria, including (a) Dataset Size; (b) image modalities; (c) AI employed, and (d) AI modality. In the RoB analysis, we considered criteria such as; (a) image modalities, (b) the objective of the study, (c) the dataset size (in the number of patients), (d) the demography of the patients, (e) feature extraction and (f) selection, (g) data preprocessing, (h) data augmentation, (i) the number of performance evaluation parameters, (j) accuracy, (k) sensitivity, (l)specificity, (m) precision, (n) AUC of the ROC, (o) the F score, (p) performance analysis metrics like the confusion matrix and ROC, (q) statistical analysis, (r) regularization, (s) number of regularization methods, (t) hardware and (u) software resources.

The novelty of our study includes determining modality with higher efficacy of several AI models in predicting the MGMT status of GBM patients. Most research employed ML algorithms to predict the MGMT status of GBM patients. A DL model needs a huge number of training instances, which makes large, high-quality medical imaging pictures challenging or impossible to produce. It is many advantages over conventional approaches with hand-crafted features, including being resistant to distortions like changes in form and having a lower computational cost. In addition, DL models have the advantage of automatically extracting features from the images. We have observed that the majority of the images used for diagnosis purposes are MRI images because the advanced MRI techniques such as diffusion tensor imaging, perfusion MR techniques such as arterial spin labeling, dynamic susceptibility contrast, MR spectroscopy technique, and dynamic contrast-enhanced imaging can aid with the morphology and function of tumors. Though CT and PET modalities are also very informative but are used up to a lesser extent. Various modalities are used to predict the MGMT status, but we have observed that modalities like CNN, Boosting, Random Forests, and Vector Machines are mostly used. The observation has been made that a combination of modalities works better than a single modality. The models using DL techniques have resulted in higher accuracies than the models using ML methods. We have found that only 73% of the studies we considered have a low bias (mean > 6.0). Despite many exemplary studies with great performance evaluation metrics, we have found that they have not focused on large datasets.

### Benchmarking table

The following benchmarking Table 2 compares some of the contributing research for MGMT promoter methylation estimation considered for the evaluation (Zhu et al. 2022; Zlochower et al. 2020; Alhasan 2021; Wu et al. 2021; Kempen et al. 2021), where 8 attributes were considered.

**Table2:** Benchmarking table

SN	Attributes	Zhu et al. 2022	Zlochower et al. 2020	Rizzo et al. (2018)	Wu et al. 2021	Kempen et al. 2021	Samartha et al. (proposed)
1	Date	Aug 2022	April 2020	November 2021	November 2021	May 2021	
2	PRISMA	×	×	✔	×	✔	✔
3	Number of studies	–	–	20	–	17	33
4	References	161	46	43	87	91	84
5	Bias Analysis	×	×	×	×	×	✔
6	Statistical Analysis	×	×	×	✔	✔	✔
7	AI Focus	ML	DL	ML and DL	ML	ML	ML and DL
8	Radio genomics	✔	×	×	✔	✔	✔

### Recommendations and challenges

High-quality ground truth data, generalizable and interpretable methodologies, and the integration of user-centric workflows are major obstacles to the promises of AI in radiology. Concerns over the "black box" character of these algorithms have waned in light of the ongoing advancement of methods, such as saliency mapping or principal component analysis, that may "unbox" the networks by examining internal algorithm feature vectors. So, it is recommended that a better mechanistic understanding of feature patterns and underlying biology will be helpful both for clinical acceptance and for improving the biological and treatment relevance of the patterns revealed by these methods.

The need for robust and thoroughly annotated data sets is AI research's biggest challenge. However, studies with relatively small sample numbers are likelier to have measurement errors. TCIA and BraTS have significantly produced consolidated, well-labelled data for glioma image processing. In contrast, non-glioma-based research has been constrained by the absence of a publicly available data set. However, most data are still isolated within various organizations and hospital systems. To increase the generalizability of an algorithm's performance across multiple imaging sites, acquisition parameters, and patient groups, more extensive and more diverse data sets are recommended (AlBadawy et al. 2018). Other approaches to enhancing data sets include statistical techniques to harmonize the data sets and to introduce more consistent data collecting via the adoption of standardized neuro-oncology imaging protocols across institutions (Ellingson et al. 2017).

Although processing, expenses, and various institutions' ethical approval processes make managing multi-institutional data, it is advised to manage it meticulously so that the radiogenomics study will turn out to be the best and most clinically trustworthy. For instance, if institutions cannot disclose their data owing to ethical concerns, they may release the AI models they have generated and test them on their cohort so that researchers can efficiently integrate the models and conduct additional analyses. Consequently, researchers could conduct their research with more reliable and applicable results (Saxena et al. 2022). Finally, when dealing with high-dimensional, small-sized datasets, the issue of ML model overfitting may be avoided using cross-validation to ensure that the test component does not interfere with the training process (Improving MGMT methylation status prediction of glioblastoma through optimizing radiomics features using genetic algorithm-based machine learning approach|Scientific Reports 2022).

## Conclusion

Here, multiple AI-based studies for MGMT promoter methylation estimation with numerous ML & DL architectures, datasets, accuracy, and other significant attributes have been presented. It is concluded that ML-based methods could be employed as filters, predictors, and classification methods to increase most cases' overall performance of the robust model. And DL-based methods demonstrated well performed for in-depth analysis of MGMT methylation estimation. An RoB analysis, considering 21 AI attributes, showed that 27% of studies belong to the high-bias category, and the remaining belong to the low-bias category. The highly biased studies have not considered all AI attributes while evaluating the radiogenomics system or may have lower proportioned values for the attributes considered. Though, there are specific challenges while implementing such AI-based methods for MGMT promoter methylation estimation. However, some promising results demonstrate that if the obstacles are carefully handled, these methods could play a vital role in the field of neuro-oncology in current clinical practice in the era of precision medicine.

## Appendix A

Based on the consensus of the experienced AI engineering team and deep literature review, we developed the scheme for the weight matrix, in which 21 AI-based attributes were for all **33** studies, thus a total of 693 attributes involved. These AI attributes are initially qualitative and then quantified by assigning a number between 0 and 1, as shown in Table 3, which is known as the weight matrix. The attributes are assigned to values ranging from **A1** to **A21** Table 3).

*Assigning weights to the attributes*

**A1** (*Image modality)*: MRI = 0.5, PET = 0.6, CT = 0.4, MRI + PET = 1, MRI + CT = 0.7, CT + PET = 0.8; **A2***(Study objective)*: classification or segmentation = 0.5, both classification and segmentation = 1; **A3** *(Dataset Size (# of patients))*: < 100 = 0.6, 101–200 = 0.7, 201–300 = 0.8, > 300 = 1; **A4***(Demographic info)*: no = 0.5, yes = 1; **A5***(Feature Extraction):*hand-crafted = 0.7 none = 0.5 automatic = 1; **A6***(Feature Selection):* no = 0.2 and yes = 1; **A7***(Pre-Processing):* No pre-processing used = 0.5 and pre-processing used = 1; **A8***(Data Augmentation):* No data augmentation = 0.5, with data augmentation (yes) = 1; **A9***(number of PE Parameters):* 5 or more parameters = 1, 4 parameters = 0.9, 3 parameters = 0.8, 2 parameters = 0.7, 1 parameters = 0.6; **A10***(Accuracy):*converted to percentage and scored between 0 and 1 (eg, 50% = 0.5 and 100% = 1); **A11***(Sensitivity):* converted to percentage and scored between 0 and 1 (eg, 50% = 0.5 and 100% = 1); **A12***(Specificity):* converted to percentage and scored between 0 and 1 (eg, 50% = 0.5 and 100% = 1); **A13***(Precision):*converted to percentage and scored between 0 and 1 (eg, 50% = 0.5 and 100% = 1); **A14***(F1 score):* converted to percentage and scored between 0 and 1 (eg, 50% = 0.5 and 100% = 1; **A15***(AUC):* Keep it as it is, if 0.99, the 0.99; **A16***(Performance Analysis Metrics):*no ROC and Confusion Matrix and Boxplot = 0.2, Only confusion matrix = 0.6,Only Boxplot = 0.7, Only ROC = 0.8, Both ROC and Boxplot = 0.9, Both ROC and Confusion Matrix = 1; **A17***(Statistical Analysis):*No statistical analysis = 0.2, Yes = 1; **A18***(Regularisation):* Absence in study = 0.2, Presence = 1; **A19***(# of Regularisation Methods):* no method = 0.2, 1-method = 0.8, 2-methods = 0.9, and 3-methods = 1; **A20***(Hardware Resources):* Information available = 1, No information = 0.5; **A21***(Software Resources):*Informationavailable = 1,Noinformation = 0.5.

1. *Image Modalities*: Understanding the pivotal role played by various image modalities in offering distinct insights into tumors and the inherent challenges they pose in constructing effective ML/DL models based on these insights, we recognize the paramount importance of image modality in our analyses.
2. *Study Objectives*: In our evaluation, we have duly considered the primary objectives of the studies. We have accorded higher scores to studies that encompass both classification and segmentation objectives, reflecting the prevalence of these objectives among the considered works.
3. *Dataset Size (Number of Patients)*: Recognizing the pivotal role of dataset size in determining the efficacy of statistical models, we have given preference to studies boasting larger datasets and substantial results.
4. *Patient Demographics*: Comprehending the significance of patient demographics in model training, we have included demography as a metric to encourage a holistic approach, encompassing factors such as patient age and gender.
5. *Feature Extraction*: To facilitate replicability, we have considered studies that explicitly detail their feature extraction procedures as they contribute to easier replication efforts.
6. *Feature Selection*: Similarly, we have prioritized studies that elucidate their feature selection methods, promoting replicability and improved results.
7. *Data Preprocessing*: Acknowledging the critical role of data preprocessing in the machine learning pipeline, we have rewarded studies that transparently describe their data preprocessing methods.
8. *Data Augmentation*: Recognizing the benefits of data augmentation in bridging data gaps, we have evaluated studies based on their utilization of data augmentation techniques.
9. *Performance Evaluation Parameters*: Given the multifaceted nature of model performance, we have favored studies employing a comprehensive set of evaluation metrics.
10. *Accuracy*: We have acknowledged the importance of accuracy as a key evaluation metric.
11. *Sensitivity*: Sensitivity, being a vital evaluation metric, has been given due consideration.
12. *Specificity*: Specificity, as an essential evaluation metric, has been duly recognized.
13. *Precision*: Precision, a crucial evaluation metric, has been accounted for.
14. *AUC of the ROC*: The area under the ROC curve, a pivotal evaluation metric for classification models, has been considered.
15. *F1 Score*: We have recognized the significance of the F1 score as a key evaluation metric.
16. *Performance Analysis Metrics*: To gain a comprehensive visual understanding of model results, we have awarded higher scores to studies employing multiple analytic metrics such as confusion matrices, ROC curves, and box plots.
17. *Statistical Analysis*: Studies that incorporate statistical analysis bolster the credibility of their models, and hence, we have acknowledged their contributions.
18. *Regularization*: We have emphasized the importance of regularization techniques in preventing overfitting, making it a relevant criterion in our assessment.
19. *Number of Regularization Methods*: Studies implementing multiple regularization methods have received higher scores for their versatility.
20. *Hardware Resources*: To enhance replicability, we have considered the disclosure of hardware resources used in the study as a criterion.
21. *Software Resources*: Similarly, the provision of information about software resources used in the study aids in replicability and has been included as a relevant metric.

## Appendix B

See Table 3.

**Table 3:** Grading scheme other AI attributes for ranking of studies

References	A1	A2	A3	A4	A5	A6	A7	A8	A9	A10	A11	A12	A13	A14	A15	A16	A17	A18	A19	A20	A21	Absolute Score	Mean	Mean*10	Study Rank
S1-Xi et al. (Xi et al. 2018)	0.5	1	0.6	1	1	1	1	0.5	1	0.8	0.9	0.8	0.9	0	0.93	1	1	1	0.8	0.5	1	17.23	0.82	8.20	1
S3- Chen et al. (Multi-label Inductive Matrix Completion for Joint MGMT and IDH1 Status Prediction for Glioma Patients 2020)	0.5	1	0.7	0.5	0.7	0.2	1	0.5	0.9	0.9	0.9	0	0.9	0.9	0.973	1	1	1	0.8	1	1	16.373	0.78	7.80	2
S32-Chen et al. (Multi-label Inductive Matrix Completion for Joint MGMT and IDH1 Status Prediction for Glioma Patients (2017)	0.5	1	0.7	1	1	1	1	0.5	0.7	0.9	0.9	0.9	0	0.9	0.9	1	1	0.2	0.2	0.5	1	15.8	0.75	7.52	3
S14- Haubold et al. (Fully Automated MR Based Virtual Biopsy of Cerebral Gliomas 2021)	0.5	1	0.8	1	0.7	1	1	0.5	1	0.8	0.8	0.8	0.8	0	0.854	1	1	0.2	0.2	0.5	1	15.454	0.74	7.36	4
S17- Patel et al.(Machine learning-based radiomic evaluation of treatment response prediction in glioblastoma 2021)	0.5	1	0.6	1	1	1	1	0.5	1	0.7	0.8	0.7	0.8	0	0.8	1	1	0.2	0.2	0.5	1	15.3	0.73	7.29	5
S8-Jiang et al. (Fusion Radiomics Features from Conventional MRI Predict MGMT Promoter Methylation Status in Lower Grade Gliomas 2019)	0.5	1	0.6	1	1	1	1	0.5	0.9	0.9	0.8	0.9	0	0	0.898	1	1	0.2	0.2	0.5	1	14.898	0.71	7.09	6
S6-Li et al. (2018)	0.5	1	0.7	1	1	1	1	0.5	0.9	0.8	0.7	0.9	0	0	0.88	1	1	0.2	0.2	0.5	1	14.78	0.70	7.04	7
S16-Chen et al. (Multi-Label Nonlinear Matrix Completion With Transductive Multi-Task Feature Selection for Joint MGMT and IDH1 Status Prediction of Patient With High-Grade Gliomas 2018)	0.5	0.5	0.6	1	0.7	1	1	0.5	0.9	0.8	0.8	0.7	0	0	0.787	0.6	1	1	0.8	0.5	1	14.687	0.70	6.99	8
S26-Kong et al. (18F-FDG-PET-based Radiomics signature predicts MGMT promoter methylation status in primary diffuse glioma 2019)	0.8	0.5	0.7	1	0.7	1	1	0.5	0.9	0.9	0.9	0.9	0	0	0.94	1	1	0.2	0.2	0.5	1	14.64	0.70	6.97	9
S31- Crisi et al.(Crisi and Filice 2020)	0.5	1	1	0.5	1	0.2	1	1	0.9	0.8	0.9	0.6	0.9	0.9	0	1	1	0.2	0.2	0.5	0.5	14.6	0.70	6.95	10
S15-Qian et al. (Qian et al. 2020)	0.5	0.5	0.6	1	0.7	1	1	0.5	0.9	0.7	0.7	0.7	0	0	0.7721	0.6	1	1	0.8	0.5	1	14.4721	0.69	6.89	11
S4-Kickingereder et al. (Kickingereder et al. 2016)	0.5	0.5	0.7	1	1	1	1	0.5	0.9	0.6	0.6	0.6	0	0	0.6	0.6	1	1	0.8	0.5	1	14.4	0.69	6.86	12
S21-Korfiatis et al. (MRI texture features as biomarkers to predict MGMT methylation status in glioblastomas 2016)	0.5	1	0.7	1	0.7	1	1	0.5	0.8	0	0.8	0.8	0	0	0.85	0.8	1	1	0.8	0.5	0.5	14.25	0.68	6.79	13
S28-Lu et al. (Machine learning-based radiomic, clinical and semantic feature analysis for predicting overall survival and MGMT promoter methylation status in patients with glioblastoma 2020)	0.5	1	0.7	1	0.7	1	1	0.5	0.7	0.7	0	0	0	0	0.979	1	1	0.2	0.2	0.5	1	12.679	0.68	6.79	14
S29- Chen et al. (Chen et al. 2018)	0.5	1	0.6	1	0.7	1	1	0.5	0.8	0.8	0.5	0.9	0	0.2	0.85	1	1	0.2	0.2	0.5	1	14.25	0.68	6.79	15
S30-Patel et al. (Patel et al. 2021)	0.5	1	0.8	0.5	1	0.2	1	1	0.9	0.9	0.9	0.9	0	0	0.93	0.6	1	0.2	0.2	0.5	1	14.03	0.67	6.68	16
S5-Le et al. (Le et al. 2020)	0.5	1	0.6	1	1	1	0.5	0.5	0.9	0.9	0.9	0.9	0	0	0.896	1	1	0.2	0.2	0.5	0.5	13.996	0.67	6.66	17
S13-Wei et al. (2019)	0.5	1	0.7	1	1	1	0.5	0.5	0.9	0.9	0.9	0.9	0	0	0.9256	0.8	1	0.2	0.2	0.5	0.5	13.9256	0.66	6.63	18
S23-Zhang et al.(Automated machine learning to predict the co-occurrence of isocitrate dehydrogenase mutations and O6 -methylguanine-DNA methyltransferase promoter methylation in patients with gliomas 2021)	0.5	1	0.7	1	0.7	1	0.5	0.5	0.9	0.9	0.8	0.9	0	0	0.951	0.6	1	0.2	0.2	0.5	1	13.851	0.66	6.60	19
S22-Kocher et al. (Kocher et al. 2020)	1	1	0.6	1	0.7	1	1	0.5	0.8	0	0.7	0.7	0	0	0.757	1	1	0.2	0.2	0.5	1	13.657	0.65	6.50	20
S27-Baine et al. (Baine et al. 2021)	0.5	1	0.6	1	0.7	1	1	0.5	0.8	0	0.7	0.9	0	0	0.89	1	1	0.2	0.2	0.5	1	13.49	0.64	6.42	21
S20-Baine et al. (The Potential Use of Radiomics with Pre-Radiation Therapy MR Imaging in Predicting Risk of Pseudoprogression in Glioblastoma Patients 2021)	0.5	1	0.6	1	0.7	1	0.5	0.5	0.8	0	0.6	0	0.6	0.6	0.82	1	1	0.2	0.2	0.5	1	13.12	0.62	6.25	22
S24-Kanas et al. (Learning MRI-based classification models for MGMT methylation status prediction in glioblastoma 2017)	0.5	1	0.6	1	0.7	1	1	0.5	0.8	0.7	0.8	0.7	0	0	0	0.6	1	0.2	0.2	0.5	1	12.8	0.61	6.10	23
S33-Do et al. (Improving MGMT methylation status prediction of glioblastoma through optimizing radiomics features using genetic algorithm-based machine learning approach 2022)	0.5	1	0.6	0.5	0.7	1	0.5	0.5	0.8	0.9	0.9	0.9	0	0	0	1	1	0.2	0.2	0.5	1	12.7	0.60	6.05	24
S12-Qian et al. (Prediction of MGMT Status for Glioblastoma Patients Using Radiomics Feature Extraction From 18F-DOPA-PET Imaging 2020)	0.8	1	0.6	1	1	1	0.5	0.5	0.8	0.8	0.8	0.7	0	0	0	0.2	1	0.2	0.2	0.5	1	12.6	0.60	6.00	25
S11-Crisi et al. (Predicting MGMT Promoter Methylation of Glioblastoma from Dynamic Susceptibility Contrast Perfusion: A Radiomic Approach 2020)	0.5	0.5	0.6	1	0.7	1	0.5	0.5	0.8	0	0.8	0.9	0	0	0.84	0.9	1	0.2	0.2	0.5	1	12.44	0.59	5.92	26
S25-Huang et al. (Radiological model based on the standard magnetic resonance sequences for detecting methylguanine methyltransferase methylation in glioma using texture analysis-PubMed 2022)	0.5	0	0.6	1	0.7	1	1	0.5	0.8	0	0.7	0.9	0	0	0.833	1	1	0.2	0.2	0.5	1	12.433	0.59	5.92	27
S7-Sohn et al. (Radiomic Analysis to Predict Histopathologically Confirmed Pseudoprogression in Glioblastoma Patients-ScienceDirect 2022)	0.5	1	1	0.5	1	0.2	0.5	1	0.8	0	0.5	1	0	0	0.761	0.6	1	0.2	0.2	0.5	1	12.261	0.58	5.84	28
S9- Lu et al. (Lu et al. 2020)	0.5	0.5	0.7	1	1	1	1	0.5	0.6	0	0	0	0	0	0	0.2	1	1	0.8	0.5	1	11.3	0.54	5.38	29
S2-Chang et al. (Radiomics-based machine learning model for efficiently classifying transcriptome subtypes in glioblastoma patients from MRI-ScienceDirect 2022)	0.5	1	0.8	1	0.7	1	1	0.5	0.6	0.9	0	0	0	0	0	0.2	1	0.2	0.2	0.5	0.5	10.6	0.50	5.05	30
S10-Hajianfar et al. (MRI-Based Deep-Learning Method for Determining Glioma MGMT Promoter Methylation Status-PubMed 2022)	0.5	1	0.6	1	1	1	0.5	0.5	0.6	0	0	0	0	0	0.78	0.7	1	0.2	0.2	0.5	0.5	10.58	0.50	5.04	31
S18-Verduin et al. (Comprehensive Genomic Subtyping of Glioma Using Semi-Supervised Multi-Task Deep Learning on Multimodal MRI|IEEE 2022)	0.5	0.5	0.7	1	0.7	0.2	1	0.5	0.6	0	0	0	0	0	0.7	0.2	1	0.2	0.2	0.5	1	9.5	0.45	4.52	32
S19-Kocher et al. (Predicting MGMT Promoter Methylation in Diffuse Gliomas Using Deep Learning with Radiomics-PMC 2022)	1	1	0.6	0.5	0.7	1	1	0.5	0.5	0	0	0	0	0	0	0.2	0.2	0.2	0.2	0.5	0.5	8.6	0.41	4.10	33

*A1* Image modality; *A2* Study objective; *A3* Dataset Size (# of patients); *A4* Demographic info; *A5* Feature Extraction; *A6* Feature Selection; *A7* Pre-Processing; *A8* Data Augmentation; *A9* number of PE Parameters; *A10* Accuracy; *A11* Sensitivity; *A12* Specificity; *A13* Precision; *A14* F1 score; *A15* AUC; *A16* Performance Analysis Metrics; *A17* Statistical Analysis; *A18* Regularisation; *A19* # of Regularisation Methods; *A20* Hardware Resources; *A21* Software Resources

## Abbreviations

AI: Artificial intelligence
ANN: Artificial neural network
AUC: Area under curve
BraTS: Brain tumor segmentation
CNN: Convolutional neural network
CT: Computer tomography
DL: Deep learning
DS: Dataset size
DSA: Digital subtraction angiography
E: Exclusion criteria
FCN: Fully convolutional network
FLAIR: Fluid-attenuated inversion recovery
GA: Genetic algorithm
GBM: Glioblastoma multiforme
I: Inclusion criteria
KNN: K-Nearest Neighbours
MGMT: O6-methylguanine-DNA methyltransferase
ML: Machine learning
MRI: Magnetic resonance imaging
NN: Neural networks
OOP: Out-of-pocket
OS: Overall Survival
PET: Positron emission tomography
PRISMA: Preferred reporting items for systematic reviews and meta-analyses
RF: Random forest
RoB: Risk-of-bias
ROI: Region of interest
SVM: Support vector machine
T1WI: T1-weighted image
T2WI: T2-weighted image
TCIA: The cancer imaging archive
TMZ: Temozolomide
WHO: World Health Organisation
XGBoost: EXtreme Gradient Boosting
